# Long-term Outcomes of Tibial Spine Avulsion Fractures After Open Reduction With Osteosuturing Versus Arthroscopic Screw Fixation: A Multicenter Comparative Study

**DOI:** 10.1177/23259671231176991

**Published:** 2023-06-20

**Authors:** Maija Jääskelä, Marco Turati, Lasse Lempainen, Nicolas Bremond, Aurelien Courvoisier, Antoine Henri, Franck Accadbled, Jaakko Sinikumpu

**Affiliations:** * Department of Pediatric Orthopaedics and Surgery, Oulu University Hospital; Research Unit of Clinical Medicine, University of Oulu; and Medical Research Center, Oulu, Finland.; † Orthopedic Department, Fondazione IRCCS San Gerardo dei Tintori, Monza, Italy.; ‡ Department of Medicine and Surgery, University of Milano-Bicocca, Monza, Italy.; § Ripoll y De Prado FIFA Medical Centre of Excellence, Madrid, Spain.; ∥ FinnOrthopaedics/Hospital Pihlajalinna, Turku, Finland.; ¶ Department of Physical Activity and Health, Paavo Nurmi Centre, University of Turku, Turku, Finland.; ＃ Transalpine Center of Pediatric Sports Medicine and Surgery, University of Milano-Bicocca, Monza, Italy.; ** Department of Paediatric Orthopaedic Surgery, Hospital Couple Enfant, Grenoble Alpes University, Grenoble, France.; †† Unité de Médecine du Sport, Centre Hospitalier Francois Mitterand de Pau, Pau, France.; ‡‡ Department of Orthopaedic Surgery, Hôpital des Enfants, Centre Hospitalier Universitaire de Toulouse, Toulouse, France.; §§ Hospital TerveysTalo, Oulu, Finland.; *Investigation performed at Oulu University Hospital, Oulu, Finland; Hospital Couple Enfant, Grenoble Alpes University, Grenoble, France; Hôpital des Enfants, Centre Hospitalier Universitaire de Toulouse, Toulouse, France; and Fondazione IRCCS San Gerardo dei Tintori, Monza, Italy*

**Keywords:** children and adolescents, tibial spine fracture, knee ligaments, treatment methods, pediatric sport medicine, general sports trauma

## Abstract

**Background:**

More information is needed regarding return to preinjury sport levels and patient-reported outcomes after tibial spine avulsion (TSA) fracture, which is most common in children aged 8 to 12 years.

**Purpose:**

To analyze return to play/sport (RTP), subjective knee-specific recovery, and quality of life in patients after TSA fracture treated with open reduction with osteosuturing versus arthroscopic reduction with internal screw fixation.

**Study Design:**

Cohort study; Level of evidence, 3.

**Methods:**

This study included 61 patients <16 years old with TSA fracture treated via open reduction with osteosuturing (n = 32) or arthroscopic reduction with screw fixation (n = 29) at 4 institutions between 2000 and 2018; all patients had at least 24 months of follow-up (mean ± SD, 87.0 ± 47.1 months; range, 24-189 months). The patients completed questionnaires regarding ability to return to preinjury-level sports, subjective knee-specific recovery, and health-related quality of life, and results were compared between treatment groups. Univariate and multivariate logistic regression analyses were conducted to determine variables associated with failure to return to preinjury level of sport.

**Results:**

The mean patient age was 11 years, with a slight male predominance (57%). Open reduction with osteosuturing was associated with a quicker RTP time than arthroscopy with screw implantation (median, 8.0 vs 21.0 weeks; *P* < .001). Open reduction with osteosuturing was also associated with a lower risk of failure to RTP at preinjury level (adjusted odds ratio, 6.4; 95% CI, 1.1-36.0; *P* = .035). Postoperative displacement >3 mm increased the risk of failure to RTP at preinjury level regardless of treatment group (adjusted odds ratio, 15.2; 95% CI, 1.2-194.9; *P* = .037). There was no difference in knee-specific recovery or quality of life between the treatment groups.

**Conclusion:**

Open surgery with osteosuturing was a more viable option for treating TSA fractures because it resulted in a quicker RTP time and a lower rate of failure to RTP as compared with arthroscopic screw fixation. Precise reduction contributed to improved RTP.

A tibial spine avulsion (TSA) fracture is often referred to as the pediatric equivalent of an anterior cruciate ligament (ACL) rupture in adults. It is most common in children aged 8 to 12 years.^8^ These injuries occur at an estimated frequency of 3 per 100,000 people.^18^ The incompletely ossified tibial eminence in children is biomechanically weaker than the native ACL fibers; hence, injury to the knee results in a bony avulsion fracture instead of a midsubstance ACL rupture.^19^ ACL avulsion in children is typically an isolated injury and has a low trauma energy level.^14^

The most common incidents behind TSA fractures are falls from bicycles, motor vehicle accidents, and sports incidents such as skiing and football.^3,16,35^ The severity of the injury usually determines the treatment; however, there is no fully agreed-on recommended treatment. The most often used scheme for categorizing TSA fractures is the Meyers and McKeever classification^21,32^:

- Type 1 represents nondisplaced or minimally displaced fractures, which are treated without surgery.
- Type 2 is a partially displaced fracture, and its management remains controversial.^4,9,13,16,35^
- Type 3 indicates a completely displaced fracture that requires reduction and fixation (Figure 1).^6,7,13,25,37^

**Figure 1.**
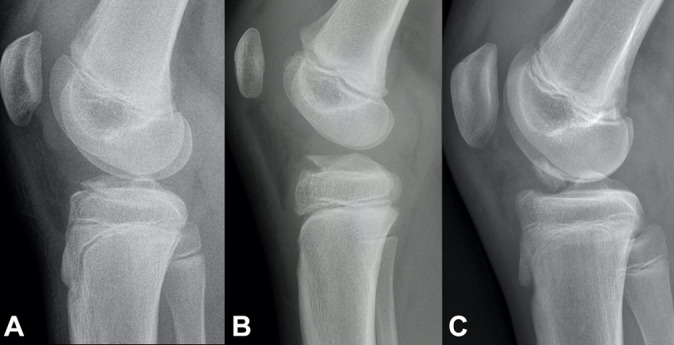
Modified Meyers and McKeever classification. (A) Type 1: nondisplaced (<3 mm). (B) Type 2: minimally displaced with intact posterior hinge. (C) Type 3: completely displaced with or without rotation.

Traditionally, open reduction with suture fixation has been performed via a knee arthrotomy. Owing to advances in knee arthroscopy, many experts prefer treating TSA fractures by performing arthroscopic reduction with internal screw fixation instead of open surgery. In comparison with osteosuturing, the screws used in screw fixation provide a more rigid fixation. However, there is no need for future hardware removal after osteosuturing. There is no consensus in the clinical or biomechanical literature to favor either suture or screw fixation in skeletally immature patients.^5,20,29^ The extant literature on the subject is heterogeneous.^5,9,11,25,29^ Ultimately, evidence for the superiority of arthroscopic fixation over open reduction with fixation has been insufficient.^5,9^

Anterior instability, which is thought to be due to traumatic elongation of the ligament rather than inadequate fixation, is one of the most frequently reported long-term findings after a TSA fracture.^1,15,34^ Malunion, nonunion, growth disturbance, pain, decreased range of motion, and arthrofibrosis, in particular, have also been confirmed as long-term sequelae.^6,7,15,33-35^ However, return to play/sport (RTP) and subjective patient-related outcomes over the long term are not widely understood.^1,17,34^

In this study, we analyzed time to preinjury-level RTP and subjective outcomes in children and adolescents who were treated for TSA fractures as a function of the treatment method: open reduction with osteosuturing versus arthroscopic reduction with internal screw fixation.

## Methods

Ethics committee approval for the study protocol was provided from the primary study center, and institutional approval was provided from every affiliated institution according to the local regulation. All study participants provided informed consent. This multicenter observational case-control study by an international research group (the European Paediatric Orthopaedic Society Sports Group; active members include M.T., F.A., and J.S.) was conducted at 4 European institutions. We queried the institutional databases for patients who had been treated for a TSA fracture via open osteosuturing or arthroscopic screw fixation at any of the 4 study centers between 2000 and 2018. Patients <16 years old at the time of injury who had a minimum 24 months of follow-up at the time of data collection (June 2020) were initially selected to participate in the study. The hospital journals and radiographs of the preliminary selected patients were reviewed to confirm their diagnosis and eligibility. The injury mechanism, fracture grade based on the Meyers and McKeever classification system, pre- and postoperative displacement, treatment method details, and the number of follow-up visits were determined.

During the study period, 173 patients with a TSA fracture were treated operatively or nonoperatively at 1 of the 4 study institutions. Of these patients, 116 met the preliminary inclusion criteria for this study. The exclusion criteria were previous ACL injury and/or reconstruction, an infectious antecedent, connective tissue disorders, or rheumatologic diseases, but there was no such patient. Patients with a subsequent severe injury to the same lower extremity with the TSA fracture—any injury requiring surgical treatment was considered severe—were also excluded. There was only 1 patient who sustained a severe lower-leg fracture within 24 months of sustaining the tibial spine injury and was thus excluded.

### Outcome Measures

A survey was mailed to the included patients between August and December 2020. This survey included patient-reported outcome instruments for knee-specific recovery and health-related quality of life, as well as questions regarding RTP ability. Patients who did not return the survey were contacted 2 more times during data collection (August-December 2020).

The patient-reported outcome instrument was selected according to the age of the patient at the time of follow-up (not at the time of injury). Knee-specific recovery was measured using the International Knee Documentation Committee (IKDC) Subjective Knee Form (scored from 0 to 100, with higher scores indicating higher levels of function).^12,17^ To determine satisfactory knee recovery, the age- and sex-matched normative data for the IKDC 2000 for men and women with no current or past knee problem were used as cutoff scores: 95.5 for men and 93.4 for women aged 18 to 24 years and 94.6 for men and 92.5 for women aged 25 to 34 years.^2^ Patients <18 years old completed the pediatric version of the IKDC (Pedi-IKDC), and the published median value of 94.6 for patients aged 10 to 18 years^24^ was used as the cutoff score for determining adequate versus impaired knee-specific recovery. Health-related quality of life was measured using the EuroQol 5-level EQ-5D (EQ-5D-5L) for patients >18 years old and the youth EQ-5D (EQ-5D-Y) for patients <18 years old.^10,36^ In part 1, patients choose responses from among 5 levels for the EQ-5D-5L (*no problems* to *extreme problems*) or from among 3 levels for the EQ-5D-Y (*no problems* to *a lot of problems*) in response to 5 categories: mobility, self-care, usual activities, pain/discomfort, and anxiety/depression. Impaired quality of life was determined as any problem (level ≥2) in any of the 5 categories. In part 2, patients record their health on the day of the survey on a visual analog scale (0 = worst, 100 = best).

The primary outcome variable was the ability to return to preinjury-level sport after sustaining the TSA fracture. Patients were asked about their preinjury sport levels: whether they were able to return to the same level at any time after the injury and, if not, whether they were able to return to a lower level of that sport. If a patient did not return to the same level, he or she was asked to provide the reason.

### Statistical Analysis

Outcomes were compared between patients who underwent open osteosuturing and those who had arthroscopic screw fixation. Descriptive statistics are presented as means, ranges, and standard deviations for normally distributed continuous variables as appropriate. Normality of the data set was investigated using the Shapiro-Wilk test. Mean values were compared with the *t* test; nonnormally distributed variables are reported as medians and were compared via the Mann-Whitney *U* test. Dichotomous variables are presented as frequencies and proportions. Differences between the proportions of the variables were analyzed using the standardized nominate deviation test, and the distribution of the variables was analyzed via the chi-square test or Fisher exact test for small sample sizes (n < 5).

The risk of impaired outcomes was analyzed through logistic regression analysis, with crude and adjusted odds ratios (ORs) presented. Given our expertise, we deduced before study initiation that the following would be associated with the risk of unsatisfactory outcomes (ie, failure to return to preinjury-level sport): older age, male sex, displacement >5 mm, postoperative displacement >3 mm, associated injuries at the time of TSA fracture, osteosutures as compared with screw fixation, and the need for reoperation during the follow-up. Therefore, regardless of whether results were significant in the univariate analysis (crude values), these potentially important variables were included in the multivariate logistic regression analysis. The threshold of statistical significance was *P* < .05, and we required that all analyses be 2-tailed and that 95% confidence intervals be used wherever possible. The statistical analyses were performed with SPSS Version 27.0 (IBM Corp) and StatsDirect Version 3.0 (StatsDirect Ltd).

## Results

### Patient Characteristics and Injury Types

Of the 116 initial study participants, 54 did not return the completed questionnaires. Thus, the final number of study participants was 61 (53% of the 116 who were treated via open reduction with osteosuture fixation or arthroscopic reduction with screw fixation). The mean ± SD follow-up time was 87.0 ± 47.1 months (range, 24-189 months).

Most of the study patients were male (n = 35; 57.4%). The mean age was 11.2 ± 2.6 years (range, 5.25-15.5 years): 11.9 ± 2.4 years for boys and 10.4 ± 2.5 years for girls at the time of injury. The predominant reasons for the sports-related TSA fractures were ski falls (57.4%), followed by bicycle or motorbike accidents (19.7%) and ball game–related accidents (13.1%). Two parameters were higher among patients who had open surgery than among those who underwent arthroscopy: preoperative displacement (7.9 vs 5.4 mm; *P* = .009) and postoperative displacement (1.3 vs 0.5 mm; *P* = .012) (Table 1).

**Table 1:** Characteristics of the Study Patients Overall and by Study Group*^a^*

	Overall (N = 61)	Open Osteosuture (n = 32)	Arthroscopic Screw (n = 29)	Difference (95% CI)	*P^b^*
Age, y	11.2 ± 2.6	10.9 ± 2.7	11.5 ± 2.3	0.6 (–0.7 to 1.9)	.371
Follow-up, mo	87.0 ± 47.1	101.4 ± 51.9	71.0 ± 35.6	30.4 (7.7 to 53.0)	**.010**
Sex					
Female	26 (42.6)	14 (43.8)	12 (41.4)	2.4 (–22.2 to 26.5)	>.999
Male	35 (57.4)	18 (56.2)	17 (58.6)		
Sport when injured					
Skiing	35 (57.4)	23 (71.9)	12 (41.4)	30.5 (5.5 to 52.1)	**.013**
Bicycle/motorbike	12 (19.7)	7 (21.9)	5 (17.2)	4.7 (–16.3 to 24.9)	.548
Ball games	8 (13.1)	2 (6.2)	6 (20.7)	14.4 (–3.0 to 33.4)	.079
Other	6 (9.8)	0 (0)	6 (20.7)	20.7 (8.7 to 38.6)	**.004**
Injury side					
Left	33 (54.1)	13 (40.6)	15 (51.7)	11.1 (–13.9 to 34.8)	.325
Right	28 (45.9)	19 (59.4)	14 (48.3)		
TSA fracture grade*^c^*					
Type 1	1 (1.6)	0 (0)	1 (3.4)	3.4 (–7.6 to 17.3)	.237
Type 2	26 (42.6)	12 (37.5)	14 (48.3)	10.8 (–14.0 to 34.4)	.324
Type 3	34 (55.7)	20 (62.5)	14 (48.3)	14.2 (–10.8 to 37.6)	.218
Preoperative imaging					
Radiograph	61 (100)	32 (100)	29 (100)	NA	NA
CT	15 (24.6)	7 (21.9)	8 (27.6)	5.7 (–16.1 to 27.7)	.570
MRI	7 (11.5)	6 (18.8)	1 (3.5)	15.3 (–1.1 to 32.7)	.315
Displacement, mm					
Pretreatment	6.7 ± 3.5	7.9 ± 3.3	5.4 ± 3.4	2.5 (0.6 to 4.3)	**.009**
Posttreatment	0.9 ± 1.3	1.3 ± 1.5	0.5 ± 0.8	0.8 (0.2 to 1.4)	**.012**
Concomitant injuries	9 (14.8)	6 (18.8)	3 (9.4)	8.4 (–10.7 to 27.1)	.315

*^a^* Data are reported as mean ± SD or No. (%) of patients unless otherwise indicated. CT, computed tomography; MRI, magnetic resonance imaging; NA, not applicable; TSA, tibial spine avulsion.

*^b^* Bold *P* values indicate statistically significant difference between study groups (*P* < .05). Standardized nominate deviation test used for proportions; *t* test used for means.

*^c^* According to Meyers and McKeever classification.^21^

### Displacement and Primary Treatment

One injury (1.6%) was classified as type 1, 26 (42.6%) as type 2, and 34 (55.7%) as type 3. One patient with a grade 1 injury was treated operatively for a related hip fracture. There were concomitant injuries in 9 (14.8%) cases, and 3 of them had >1 concomitant injury. The majority of these concomitant injuries (n = 11; 84.6%) were treated operatively. In total, there were 2 medial and 3 lateral meniscal tears, 3 cartilage injuries to the femur or tibiae, 2 tibial lateral condyle fractures, 1 medial collateral ligament tear, 1 hip fracture, and 1 clavicle fracture. Primary magnetic resonance imaging was performed for 7 (11.5%) patients: significantly more for patients undergoing the open method (18.8%) than arthroscopic treatment (3.5%) (Table 1).

### Return to Preinjury Level of Sport

RTP at the preinjury level was achieved by a majority of the patients (n = 46; 75.4%). However, 6 patients were unable to return to their sports at all: 3 experienced pain, 1 had restricted movement, and 2 were encumbered by pain and restricted motion. At the time of the final follow-up (24-189 months after injury), 9 patients were able to return to their preinjury sports at a lower level. Their reported reasons were fear (n = 4), pain (n = 4), and instability (n = 2).

There was no difference in RTP rate between the 29 patients (90.6%) who underwent open reduction with osteosuturing and the 26 (89.7%) who underwent arthroscopic screw fixation (*P* = .899). Neither was there any difference in preinjury-level RTP rate between the 26 patients (81.2%) who had open surgery and the 20 (69.0%) who were treated arthroscopically (*P* = .255).

The mean period of immobilization was 4.5 ± 0.7 weeks for both study groups, and the median ± SD time to full RTP was 13.0 ± 33.0 weeks after injury for the entire study population. Patients treated via arthroscopy with screw fixation took a longer time to RTP than those treated via open surgery (median ± SD, 21.0 ± 43.4 vs 8.0 ± 2.7 weeks; *P* < .001). Patients treated arthroscopically visited out-hospital clinics more frequently during follow-up than those who underwent open surgery (mean ± SD, 4.0 ± 2.4 vs 1.9 ± 1.1; *P* < .001) (Table 2).

**Table 2:** Treatment Details and Outcomes Overall and by Study Group*^a^*

	Overall (N = 61)	Open Osteosuture (n = 32)	Arthroscopic Screw (n = 29)	Difference (95% CI)	*P^b^*
Primary fixation approach	NA	32 (52.5)	29 (47.5)	5 (–12.8 to 22.3)	.593
Immobilization time, wk	4.5 ± 0.7	4.5 ± 0.6	4.5 ± 0.7	NA	NA
No. of follow-up visits	2.9 ± 2.1	1.9 ± 1.1	4 ± 2.4	2.1 (1.1 to 3.1)	**<.001**
Reoperation by primary fixation	9 (14.8)	3 (9.4)	6 (20.7)	11.3 (–7.2 to 30.8)	.470
Time to return to sport, wk	13.0 ± 33.0	8.0 [8-12]*^c^*	21.0 [12-36.3]*^c^*	13.0*^d^*	**<.001**
Return to sport					
At preinjury level	46 (75.4)	26 (81.2)	20 (69.0)	12.2 (–9.7 to 33.9)	.255
At a lower level	9 (14.8)	3 (9.4)	6 (20.7)	11.3 (–7.2 to 30.8)	.180
Unable to return	6 (9.8)	3 (9.4)	3 (10.3)	0.9 (–18.7 to 15.8)	>.999
Knee-specific recovery: IKDC/Pedi-IKDC (max: 100)	90.9 ± 12.7	93.1 ± 13.5	90.4 ± 14.5	2.7 (–4.6 to 9.9)	.467
Health-related quality of life: EQ-5D-5L/EQ-5D-Y*^e^*					
VAS of health today (max: 100)	88.4 ± 11.3	85.4 ± 13.4	89.4 ± 10.5	4.0 (–2.2 to 10.1)	.200
Mobility					
No problems (level 1)	54 (88.5)	30 (93.8)	24 (82.8)	11.0 (–5.9 to 29.5)	.144
Any problems (level ≥2)	7 (11.5)	2 (6.2)	5 (17.2)		
Self-care					
No problems (level 1)	60 (98.4)	32 (100)	28 (96.6)	3.4 (–7.6 to 17.3)	.238
Any problems (level ≥2)	1 (1.6)	0 (0)	1 (3.4)		
Usual activities					
No problems (level 1)	54 (88.5)	31 (96.9)	23 (79.3)	17.6 (1.7 to 36.9)	**.025**
Any problems (level ≥2)	7 (11.5)	1 (3.1)	6 (20.7)		
Pain/discomfort					
No problems (level 1)	40 (65.6)	24 (75.0)	17 (58.6)	16.4 (–7.4 to 38.9)	.187
Any problems (level ≥2)	21 (34.4)	8 (15.0)	12 (41.4)		
Anxiety/depression					
No problems (level 1)	50 (82.0)	28 (87.5)	22 (75.9)	11.6 (–8.2 to 32.0)	.238
Any problems (level ≥2)	11 (18.0)	4 (12.5)	7 (24.1)		
Overall score					
No problems (level 1)	35 (57.4)	22 (68.8)	13 (44.8)	24 (–1.1 to 46.3)	.059
Any problems (level ≥2)	26 (42.6)	10 (32.2)	16 (55.2)		

*^a^* Data are reported as mean ± SD or No. (%) unless otherwise indicated. IKDC, International Knee Documentation Committee; EQ-5D-5L/Y, Euroqol 5 level Health-related questionaire 5L for >18 years old, Y for <18 years old; IQR, interquartile range; max, maximum; NA, not applicable; Pedi-IKDC, pediatric version (<18 years old) of International Knee Documentation Commitee (IKDC) subjective Knee Form; VAS, visual analog scale.

*^b^* Bold *P* values indicate statistically significant difference between study groups (*P* < .05). Standardized nominate deviation test used for proportions; *t* test used for means; and Mann-Whitney *U* test used for nonnormally distributed values (median and IQR).

*^c^* Median [IQR].

*^d^* 95% CI not applicable for the difference of medians.

*^e^* EQ-5D-5L levels: 1, no problems; 2, slight problems; 3, moderate problems; 4, severe problems; 5, extreme problems/unable to do. EQ-5D-Y levels: 1, no problems; 2, some problems; 3, a lot of problems.

Regression analyses indicated that the following were associated with a higher risk of failure to RTP at the preinjury level: arthroscopic screw fixation (adjusted OR, 6.4; 95% CI, 1.1-36.0; *P* = .035) and postoperative displacement >3 mm (adjusted OR, 15.2; 95% CI, 1.2-194.9; *P* = .037). Preoperative displacement, associated injuries, reoperation, sex, and age showed no correlation with an increased or decreased risk of ending a sports career (Table 3).

**Table 3:** Risk Factors for Inability to Return to Preinjury Level of Sport After Tibial Spine Fractures (N = 61)*^a^*

	Crude		Adjusted	
	OR (95% CI)	*P^b^*	OR (95% CI)	*P*
Age	1.3 (1.0-1.7)	.057	1.1 (0.8-1.5)	.628
Male sex	2.9 (0.8-10.3)	.105	6.1 (1.0-37.2)	.052
Displacement				
Preoperative >5 mm	0.9 (0.3-2.9)	.839	1.2 (0.3-5.7)	.776
Postoperative >3 mm	7.2 (1.2-44.0)	**.033**	15.2 (1.2-194.9)	**.037**
Associated injuries	2.7 (0.6-11.5)	.189	3.3 (0.4-26.0)	.267
Screw fixation	1.5 (0.8-2.7)	.168	6.4 (1.1-36.0)	**.035**
Reoperation	0.7 (0.1-3.1)	.601	1.3 (0.2-10.7)	.824

*^a^* Bold *P* values indicate statistical significance (*P* < .05). OR, odds ratio.

*^b^* Standardized nominate deviation test used for proportions; *t* test used for means.

### Subjective Satisfaction and Quality of Life

The mean IKDC/Pedi-IKDC score was 90.9 ± 12.7, with no significant differences between the open osteosuturing group and the arthroscopic screw fixation group (93.1 ± 13.5 vs 90.4 ± 14.5; *P* = .467). At final follow-up, there was no statistically significant difference in subjective overall health-related quality of life between the open osteosuturing and arthroscopic screw fixation groups (85.4 ± 13.4 vs 89.4 ± 10.5; *P* = .200).

Patients who needed reoperation had a 19-fold risk of reporting unsatisfactory outcomes on the IKDC (adjusted OR, 19.0; 95% CI, 1.8-203.0; *P* = .015) (Tables 2 and 4).

**Table 4:** Risk of Unsatisfactory Subjective Knee-Specific Recovery and Impaired Quality of Life After Tibial Spine Fracture (N = 61)*^a^*

	Impaired Knee-Specific Recovery*^b^*				Impaired Quality of Life*^c^*			
	Crude		Adjusted		Crude		Adjusted	
	OR (95% CI)	*P*	OR (95% CI)	*P*	OR (95% CI)	*P*	OR (95% CI)	*P^d^*
Age	1.1 (0.9-1.4)	.259	1.1 (0.8-1.4)	.572	1.0 (0.8-1.2)	.771	0.9 (0.7-1.2)	.470
Male sex	1.2 (0.4-3.4)	.773	1.6 (0.4-6.5)	.520	1.7 (0.6-5.1)	.278	2.3 (0.6-8.9)	.237
Displacement								
Preoperative >5 mm	0.4 (0.1-1.1)	.081	0.4 (0.1-1.5)	.157	0.8 (0.3-2.4)	.708	1.1 (0.3-4.0)	.874
Postoperative >3 mm	1.8 (0.3-10.0)	.480	1.2 (0.1-11.4)	.884	0.2 (0-2.1)	.206	0.2 (0.0-3.7)	.298
Associated injuries	1.5 (0.3-6.2)	.601	0.7 (0.1-5.6)	.714	0.6 (0.1-2.8)	.544	0.6 (0.1-4.5)	.649
Screw fixation	1.7 (0.6-4.8)	.353	1.0 (0.3-4.0)	.979	2.7 (1.0-7.7)	.062	2.5 (0.7-9.0)	.160
Reoperation	21.1 (2.4-184.8)	**.006**	19.0 (1.8-203)	**.015**	3.2 (0.7-14.3)	.127	5.3 (0.8-36.1)	.090

*^a^* Bold *P* values indicate statistical significance (*P* < .05). IKDC, International Knee Documentation Committee; EQ-5D-5L/Y, Euroqol 5 level Health-related questionaire 5L for >18 years old, Y for <18 years old; OR, odds ratio; Pedi-IKDC, pediatric version (<18 years old) of International Knee Documentation Commitee (IKDC) subjective Knee Form. .

*^b^* By IKDC/Pedi-IKDC. Impaired recovery defined as score lower than age- and sex-matched normative scores.

*^c^* By EQ-5D-5L/EQ-5D-Y. Impaired quality of life categorized dichotomously (no problems vs any problems).

*^d^* Standardized nominate deviation test used for proportions; *t* test used for means.

### Reoperation

Nine patients (14.8%) required a secondary operation. One patient treated primarily via open osteosuturing sustained a communitive fracture and required ACL reconstruction at the later stage of the study. Two patients who were treated via the open technique with osteosutures had impaired range of motion after the primary operation, which was not resolved via training or physical therapy. One of these 2 patients underwent manipulation while under general anesthesia, and the other was operated on after arthroscopic removal of adhesions and then underwent manipulation. Of 6 patients who were treated primarily via arthroscopic screw fixation and needed reoperation, 3 experienced postoperative pain. Removal of the screws resolved these symptoms. Two patients, treated primarily via arthroscopic fixation, presented with instability, necessitating ACL reconstruction. One patient experienced a fracture displacement despite the initial arthroscopic screw fixation, and subsequent screw fixation was performed (Table 2).

## Discussion

The main finding of this comprehensive multicenter study was that satisfactory long-term outcomes after TSA fracture, in terms of RTP and subjective recovery scores, were achieved in both treatment groups after the data were adjusted for injury severity, age, and sex. The majority of the patients (75%) in this study were able to return to their preinjury levels of sport, which is an important and encouraging report for junior athletes who are highly likely to sustain an acute TSA fracture. Furthermore, there were good subjective long-term outcomes after open and arthroscopic surgery, which support the notion that surgeons may choose between these approaches based on their preference and competence. Nevertheless, when the 2 surgical approaches were compared, we found that open surgery with osteosuturing was associated with a quicker RTP time than the other method. In addition, open surgery with osteosuturing more frequently culminated in the patient returning to sport at the preinjury level than what was observed with arthroscopic screw fixation. These results, in particular, strengthen the evidence that open fixation with osteosutures is still a valid option for treating TSA fractures, regardless of the concrete advantages of arthroscopic knee surgery.

Most patients recover well after a TSA fracture.^7,23,26-28,30^ Stallone et al^30^ reported good outcomes after operative and nonoperative care, although their study population was small and the majority of the participants were treated nonoperatively (23 vs 16). Furthermore, 78% of their patients returned to their previous levels of play in sports, and the mean Pedi-IKDC score was 96.4 ± 5.7. In contrast, the mean IKDC score in our study was slightly lower (90.9 ± 12.7); however, the proportion of patients who returned to their previous levels of play in sports in the Stallone et al study (78%) is close to that achieved in our study (75.4%), even though all patients in this study were treated operatively. In a small series, 10 patients treated via arthroscopic reduction with fixation were followed up for 7 years, and they were all able to RTP at their preinjury levels with full range of motion.^26^ Nevertheless, because of the small number of participants in the series, only weak conclusions could be reached.

In this study, just 2 patients experienced such severe instability that they were not able to return to their previous levels of sport. One of these 2 patients was treated by open osteosuturing and the other by arthroscopic screw fixation. Both patients were still able to continue playing the same sports but at a lower national level. Patients who underwent reoperation had lower IKDC scores but experienced no further impediments to returning to their previous levels of play in sports. In the literature, pain and fear of reinjury have been the most common patient-reported reasons for failing to return to preinjury-level play,^30^ which is consistent with the findings of this research.

It remains unclear which surgical approach is best for TSA fracture repair. In this study, children treated by arthroscopic surgery with screw fixation had a longer RTP than those treated by open osteosuturing. To our knowledge, the extant literature on the probable association between the surgical method and the RTP time after a TSA fracture is weak. However, it must be acknowledged that permission to RTP may be dependent on the outlook of the arthroscopic surgeons, which will differ among them, while some experts may stress the importance of overall recovery before permitting free exercise based on such particulars as quadriceps muscle strength and function, proprioception, and repair of the dynamic knee valgus.^31^ Because of the study design, the postoperative rehabilitation of the patients could not be adjusted for. Furthermore, in this study, more patients treated via arthroscopic screw fixation underwent reoperation (20.7%) than those treated via open osteosuturing (9.4%). The removal of the implants was a usual procedure, which is reasonable. The findings of this study agree with those of a study by Callahan et al,^5^ who reported that patients treated via screw fixation had an almost 3-fold higher risk of reoperation than those who underwent osteosuture fixation. Secondary removal operation may have been one explanation for the delayed RTP in the screw fixation group in this research.

### Strengths and Limitations

The strengths of this study are its multicenter design, international scope, and long follow-up time. A further strength of this study is that 2 of most essential surgical procedures were selected for comparison: open reduction with osteosuturing and arthroscopy with screw fixation. The patients were evaluated using validated questionnaires. However, greater study is needed in the future, given that the number of patients who were completely unable to return to their preinjury sports was no more than 6.

The main limitation of this study is its retrospective design; furthermore, no randomization was performed, and the treatment approach for each case was independently decided by the treating surgeon. Another limitation is that, given the study design, no clinical examination was performed during the follow-up and the participation rate was not more than 53%. In addition, there may have been individual variations in the overall rehabilitation and recovery program for muscle strengthening and balance training. Because of limited hospital records on postoperative rehabilitation, the results of this study could not be adjusted for the quality or intensity of rehabilitation. Another limitation is that several other fixation methods typically used in TSA fracture repair were not included for analysis in this study because of the small number of these cases: isolated patients treated via open reduction with screw or K-wire fixation and arthroscopic osteosuturing or K-wire fixation were excluded in adherence to the research plan. It is additionally worth recognizing that only 2 cases with clinically significant arthrofibrosis were found in this study. There may have been more patients with decreased range of motion who were not identified because the postoperative range of movement was not systematically evaluated. However, this is unlikely because arthrofibrosis is a considerable handicap, and such a clinically significant decrease in range of motion would have been reported during follow-up. It is possible that routine removal of the implants proceeded differently between the experts who performed the operations and the institutions at which they were performed, which may have affected the incidence of secondary operations. Another limitation is that there were few concomitant intra-articular injuries among the patients (14.8%). In light of the existing literature on this issue, this may have occurred because of the low rate of primary magnetic resonance imaging scans performed on the participants.^22,26^ Finally, a weakness of the study was that it focused on the patients’ return to their preinjury levels of sport; the potential progress in their sport careers after TSA was not studied and would be the aim of the future research.

## Conclusion

We found that good outcomes were achieved after a TSA fracture after open osteosuturing and arthroscopic screw fixation when the results were adjusted for severity of the injury, sex, and age. Open osteosuturing was associated with a quicker RTP time and a lower rate of failure to return to preinjury level of sport as compared with arthroscopic screw fixation. Higher postoperative displacement increased the risk of failure to return to preinjury sport level.
